# Association of Vitamin D Receptor Polymorphisms With Activity of Acromegaly, Vitamin D Status and Risk of Osteoporotic Fractures in Acromegaly Patients

**DOI:** 10.3389/fendo.2019.00643

**Published:** 2019-09-24

**Authors:** Aleksandra Jawiarczyk-Przybyłowska, Jowita Halupczok-Żyła, Katarzyna Kolačkov, Łukasz Gojny, Agnieszka Zembska, Marek Bolanowski

**Affiliations:** Department of Endocrinology, Diabetes and Isotope Therapy, Wroclaw Medical University, Wrocław, Poland

**Keywords:** acromegaly, polymorphisms, vitamin D receptor, osteoporotic fracture, TBS

## Abstract

**Introduction:** The vitamin D receptor *(VDR)* gene is one of the most widely studied tumorigenesis-related genes. The primary objective of this study was assessment of possible roles of *VDR* gene polymorphisms in acromegaly, with regard to the activity of the disease and compared them with a control group. Furthermore, we have assessed the associations between these polymorphisms with vitamin D status as well as with TBS (trabecular bone score) and risk for osteoporotic fracture in acromegaly patients.

**Materials and Methods:** We studied 69 patients with acromegaly and 51 healthy controls (CG). Acromegaly patients were divided into three subgroups on the basis of disease activity (AA, active acromegaly; CD, controlled disease; CA, cured acromegaly). In all patients, blood samples were obtained to assess the hormonal and metabolic status as well as genetic analysis. *VDR* polymorphisms were determined by means of two methods, Polymerase Chain Reactions (PCR) and minisequencing (SNaPshot).

**Results:** Genotype frequencies for *VDR* ApaI, TaqI, BsmI, and FokI polymorphisms did not deviate significantly from Hardy-Weinberg equilibrium (HWE) in the acromegaly group as well as in the control group. There was no statistically significant difference in distributions of these four VDR genotypes between acromegaly patients and the control group. This study revealed statistically significant negative correlation between risk of major osteoporotic fractures and genotypes tt (*TaqI*), aa (*ApaI*) and bb (*BsmI)* in acromegaly groups. Furthermore, the negative correlations were observed between TBS and risk for major osteoporotic fractures and hip fractures.

**Conclusions:** Our study suggests that tt (*TaqI*), aa (*ApaI*) and bb (*BsmI*) of *VDR* gene may be associated with better bone quality and microarchitecture (higher TBS), which lead to a lower risk of osteoporotic fractures in acromegaly patients. TBS may be a useful tool for predicting risk of fractures in acromegaly patients.

## Introduction

Acromegaly is a rare disease, characterized by an excessive secretion of growth hormone (GH), and consequently the insulin like growth factor 1 (IGF-1) (1). The main reason for acromegaly is GH-producing pituitary adenomas. These monoclonal tumors can occur sporadically or in a familial setting. Acromegaly can be a part of a syndromic disease appearing concomitantly with other endocrine tumors, such as in MEN1, MEN4, Carney complex, McCune-Albright and SDHx-related pituitary adenomas or being a part of familiar isolated pituitary adenoma (FIPA) in aryl hydrocarbon receptor interacting protein (*AIP*) or *GPR101* (G-protein coupled receptor 101) mutation positive and negative cases (2, 3).

The vitamin D receptor *(VDR)* is encoded by a large gene located on the long arm of chromosome 12 (locus 12q13.11) and it forms heterodimer with retinoid X receptors (RXRs). It consists of a promoter, six regulatory sequences and seven exons encoding six protein domains. Both VDR and RXRs are the members of the steroid nuclear receptor superfamily (4) and RXRs binding is important for transcription activation of VDR (5). There are many polymorphisms of the *VDR* gene described in the SNP database (http://www.ncbi.nlm.nih.gov/snp), however, only a few of them have a phenotypic effect, mainly related to the regulation of the level of *VDR* gene expression (6–8). The polymorphisms most widely focused on are four named after restriction enzymes that originally served to identify them. Those are: *FokI* (rs10735810)—located in the coding part, in the START codon—the polymorphic form which results in shortening by three amino acids; and *BsmI* (rs1544410), *ApaI* (rs7975232), *TaqI* (rs731236)—located in the sequence of the 3′-UTR sequence responsible for the stability of the resulting mRNA. Polymorphic forms of the receptor are characterized by a change in expression level. It may result in a decrease (in a case of decreased expression) or an increase (in a case of overexpression) of the effects of vitamin D in specific cells. *FokI* polymorphism appears to be functional. The 424 aa *VDR* variant (F allele) is more active than the 427 aa variant (f allele) and leads to higher transcriptional and functional activities (6). The vitamin D receptor gene is one of the most widely studied tumorigenesis-related genes. The occurrence of many different types of tumors has been associated with *VDR* polymorphisms.

The aim of the study was to assess possible roles of *VDR* gene polymorphisms in acromegaly, with regard to the activity of the disease in comparison to controls. Furthermore, we have assessed the associations between these polymorphisms with vitamin D status, calcium, magnesium, phosphate homeostasis, and trabecular bone score (TBS) derived from bone mineral density (BMD), as well as with risk for osteoporotic fracture in acromegaly patients.

## Materials and Methods

The study group included 69 acromegaly patients (43 females and 26 males, mean age 60.32 ± 11.26 years) and 51 controls (31 females and 20 males, mean age 56.71 ± 11.2 years). All participants were recruited from the Department of Endocrinology, Diabetes and Isotope Therapy, Wroclaw Medical University. The Polish recommendations on diagnostic and therapeutic management of acromegaly were used to divide the study group (SG) into 3 subgroups (active acromegaly – AA, controlled disease – CD and cured acromegaly – CA) considering clinical findings and biochemical evaluation (GH and IGF-1 concentration) (9). The patients with IGF-1 higher than the sex- and age-matched normative reference values and nadir GH above 0.4 μg/L (ng/mL) during the oral glucose tolerance test (OGTT) were recruited to the AA group (9 subjects). Four of patients had acromegaly *de novo*, before operation. The patients, who after failed surgery were receiving long-acting somatostatin analogs, were assigned to the CD group (39 subjects). All patients in the CD group showed normal IGF-1 values (sex- and age-matched), and 19 subjects had random GH below 1.0 μg/L (ng/mL). The patients who had undergone successful surgical treatment and had both normal IGF-1 values and nadir GH below 0.4 μg/L (ng/mL) during the OGTT were allocated to the CA group (21 subjects). The control group (CG) comprised 51 individuals with no clinical symptoms of acromegaly. The controls were matched to acromegaly patients with age and sex. The patients in the AA and the CD groups were treated with long-acting somatostatin analogs. In the AA group, two subjects received 40 mg of long-acting octreotide, one subject received 30 mg of long-acting octreotide and two patients received 120 mg of long-acting lanreotide, administered every 28 days intramuscularly or subcutaneously. In the CD group, 14 patients were treated with 120 mg of long-acting lanreotide and 16 subjects received long-acting octreotide, four patients 40 mg/dose, six patients 30 mg/dose, three patients 20 mg/dose and three patients 10 mg/dose. In this group, nine subjects were treated with long-acting pasireotide: eight subjects 40 mg/dose and one subject 60 mg/dose. Among the study group, 13 acromegaly patients required the hydrocortisone, 27 the L-thyroxin and one subject testosterone replacement therapy. In the CG group, nine patients were treated with L-thyroxin replacement therapy. Among acromegaly group, supplementation of vitamin D was applied in 20 subjects and three subjects received anti-resorptive therapy (bisphosphonate). In CG, three patients received supplementation of vitamin D, while no subject was treated with bisphosphonate. We created three classifications for the purpose of the study. The first was done on the basis of the activity of the disease (AA, CA, CD, CG). The second division was used to analyze the differences between the AA group, patients with both cured and controlled disease (CA + CD) and the CG. The third classification compared the patients with acromegaly (AA + CA + CD) and the CG.

The local Bioethics Committee approved the protocol of the study. All subjects gave written informed consent in accordance with the Declaration of Helsinki.

The GH and IGF-1 concentrations were assayed using a chemiluminescence immunometric assay (Immulite 2000, Siemens, USA). IGF-1 levels were also expressed in relation to the upper limit of normal (ULN) for age (IGF-1 x ULN) (patient's IGF-1 concentration divided by IGF-1 upper reference range limit matched for age). Vitamin D was assessed by chemiluminescence immunometric assay (Architect i1000, Abbott, USA), LOD (limit of quantitation LOQ: 2.4 ng/mL). The following ranges of 25(OH)D concentrations indicating vitamin D deficiency <20 ng/mL, suboptimal status 20–30 ng/mL, optimal status 30–70 ng/mL were used. Serum calcium, inorganic phosphate, magnesium and alkaline phosphatase were measured using colorimetric assays on an Architect c4000 (Abbott Laboratories, USA). References range were as follows: calcium 8.4–10.2 mg/dL, inorganic phosphate 2.3–4.7 mg/dL, magnesium 1.6–2.6 mg/dL, alkaline phosphatase 50–150 IU/L. Glucose was studied by enzymometric assay using Architect c4100 (Abbott Laboratories, USA), normal range 65–99 mg/dL. In addition, oral glucose tolerance test (OGTT, 75 g) was performed in acromegaly patients. Plasma GH, glucose and insulin concentrations were measured at 0, 60, and 120 min after oral administration of 75 g glucose. The BMD of the lumbar spine (L1–L4) and femoral neck was measured using the dual-energy X-ray absorptiometry (DXA) method by Hologic DPX densitometer. Risk of major osteoporotic fracture and hip fracture were calculated by FRAX (http://www.shef.ac.uk/FRAX). All TBS values were analyzed using the TBS iNsight software, version 3.0.3.0 (Med-Imaps, Pessac, France) using spine DXA files from the database.

The genomic DNA was isolated from peripheral blood leukocytes in accordance to the protocol of a commercial DNA isolation kit (QIAamp DNA Mini Kit, Qiagen, Hilden, Germany). Genotyping of the *VDR* polymorphisms: *FokI* (rs10735810) *BsmI* (rs1544410), *ApaI* (rs7975232), and *TaqI* (rs731236) were performed by means of two methods: Polymerase Chain Reactions (PCR) and minisequencing (SNaPshot). Specific fragments of the *VDR* gene containing four polymorphic sites were amplified using the TaKaRa Taq DNA Polymerase Amplification Kit (Takara Bio Inc., Shiga, Japan) in the presence of primers, described by Lins et al. (10). Both PCR fragments containing two polymorphic sites: *ApaI* and *TaqI* were assessed by one pair of primers (Table 1).

**Table 1:** Primer sequences for PCR reaction harboring the four *VDR* polymorphisms.

**Polymorphism**	**Forward PCR primer (5^**′**^-3^**′**^)**	**Reverse PCR primer (3^**′**^-5^**′**^)**	**Fragment size**
*FokI*	GGCCTGCTTGCTGTTCTTAC	TCACCTGAAGAAGCCTTTGC	174 [bp]
*BsmI*	CCTCACTGCCCTTAGCTCTG	CCATCTCTCAGGCTCCAAAG	209 [bp]
*ApaI*	CTGCCGTTGAGTGTCTGTGT	TCGGCTAGCTTCTGGATCAT	242 [bp]
*TaqI*			

The PCR reactions were carried out in final volume of 20 μL containing: 10 μM of each primer, 1 × PCR buffer containing 1.5 mM MgCl_2_, 200 μM dNTPs, 2 units of Taq polymerase and 200 ng of genomic DNA. The PCR reaction was performed using T-Personal Thermocycler (Biometra GmbH, Göttingen, Germany), starting with an initial denaturation step of 5 min at 95°C, followed by 35 cycles of 30 s denaturation at 95°C, 30 s annealing at 58°C and 60 s extension at 72°C, ended by a final extension for 10 min at 72°C. The post-PCR products were purified from excess primers and nucleotides by mixture of SAP and ExoI enzymes (Thermo Fisher Scientific, Waltham, MA, USA). The identification of gene polymorphisms was performed in a multiplex reaction in the presence of primers described by Lins et al. (10) (Table 2), according to the protocol of an ABI PRISM^®^ SNaPshot™ Multiplex Kit (Thermo Fisher Scientific, Waltham, MA, USA).

**Table 2:** Single-base extension primers for SNaPshot multiplex reaction.

**Polymorphism**	**Single-base extension primer**	**Primer size**
*FokI*	(T)_31_GCTGGCCGCCATTGCCTCC	50 [bp]
*BsmI*	(T)_21_CAGAGCCTGAGTATTGGGAATG	43 [bp]
*ApaI*	(T)_12_GTGGTGGGATTGAGCAGTGAGG	34 [bp]
*TaqI*	(T)_9_ GCGGTCCTGGATGGCCTC	27 [bp]

SNaPshot reaction was carried out for 25 cycles consisted of 10 s denaturation at 96°C, 5 s annealing at 50°C and 30 s extension at 60°C. Samples were electrophoresed on ABI PRISM^®^ 3100 Genetic Analyzer (Thermo Fisher Scientific, Waltham, MA, USA) and analyzed by GeneMapper^®^ Software v. 4.0 (Thermo Fisher Scientific, Waltham, MA, USA).

Statistical analysis was performed using GraphPad Prism version 8.0.0 for Mac OS (GraphPad Software, San Diego, California USA). Variables were presented as mean ± standard deviation. Depending on data distribution, the associations in means between groups were analyzed by Student's *t*-test or ANOVA. Mann-Whitney or Kruskal-Wallis tests were also used. Spearman's coefficient was applied in terms on bivariate correlations. 95% of confidence intervals was used, and the threshold for significance was *p* < 0.05. The differences in the frequencies of the VDR gene polymorphism was calculated using Chi-square test, the Hardy-Weinberg equilibrium (HWE) was tested using with the HWE Calculator (http://scienceprimer.com/hardy-weinberg-equilibrium-calculator).

## Results

The general characteristics of the study group and the control group are presented in Table 3. There was no statistical difference in general characteristics between the groups, regardless of used classification. Genotype distributions for *VDR ApaI, TaqI, BsmI*, and *FokI* polymorphisms did not deviate significantly from HWE in acromegaly group as well as in control group (*p* > 0.05).

**Table 3:** Characteristics of acromegaly patients and the control group.

**Group**	**Number**	**Age (years)**	**Body mass (kg)**	**Height (m)**	**BMI (kg/m^**2**^)**
AA	9	60.1 ± 10.9	92.2 ± 13.56	1.73 ± 0.13	30.8 ± 4.9
CD	39	61.0 ± 11.0	81.4 ± 16.1	1.67 ± 0.9	29.1 ± 4.5
CA	21	57.7 ± 11.7	87.1 ± 25.9	1.62 ± 0.2	28.7 ± 6.7
CG	51	56.7 ± 11.2	78.7 ± 14.2	1.68 ± 0.8	27.6 ± 4.0
AA+CD+CA	69	60.3 ± 11.2	84.5 ± 19.5	1.66 ± 0.15	29.2 ± 5.2

*AA, active acromegaly; CD, controlled disease; CA, cured acromegaly; CG, control group*.

Four genetic variants of the *VDR* were analyzed in this study. The distribution of vitamin D receptor *(VDR)* genotypes and minor allele frequency (MAF) in acromegaly and control groups are shown in Table 4. There was no statistically significant difference in frequencies of these four VDR genotypes between acromegaly patients and the control group.

**Table 4:** Distribution of vitamin D receptor genotypes and minor allele frequency (MAF) in acromegaly and control group.

**SNP**	**Acromegaly group =69 (n, %)**	**Control group *n* = 51 (n, %)**
***TaqI*** **(rs731236)**		
TT	32 (46.37%)	20 (39.21%)
Tt	26 (37.68%)	22 (43.13%)
tt	11 (15.94%)	9 (17.64%)
x^2^	1.981	0.462
*p*-value	0.159	0.497
MAF	0.35	0.39
***BsmI*** **(rs1544410)**		
BB	32 (46.7%)	20 (39.21%)
Bb	26 (37.68%)	21(41.17%)
bb	11 (15.94%)	10 (19.60%)
x^2^	1.981	1.051
*p*-value	0.159	0.305
MAF	0.35	0.4
***ApaI*** **(rs7975232)**		
AA	17 (24.63%)	15 (29.41%)
Aa	35 (50.72%)	24 (27.05%)
aa	17 (24.63%)	12 (23.52%)
*x*^2^	0.014	0.157
*p*-value	0.904	0.692
MAF	0.5	0.47
***FokI*** **(rs10735810)**		
FF	27 (39.13%)	18 (35.29%)
Ff	32 (46.37%)	24 (47.05%)
ff	10 (14.49%)	9 (17.64%)
*x*^2^	0.011	0.042
*p*-value	0.917	0.838
MAF	0.38	0.41

*x^2^, Chi-squared value; p, Chi-squared test p-value; MAF, minor allele frequency*.

The mean 25(OH)D3 level was similar in all tested groups. The correlation between 25(OH)D3 levels and parameters of calcium, magnesium and phosphate homeostasis, as well as risk for osteoporotic fractures, was not found (data not shown).

We did not observe any statistically significant difference of TBS values between acromegaly groups and control group, regardless of the division into the groups.

In the AA group, negative correlation was shown between genotypes Ff *(FokI)* and femoral neck BMD (*r* = 0.63, *p* = 0.04). Furthermore, negative correlation was also observed between this genotype Ff *(FokI)* and TBS (*r* = 0.86, *p* = 0.007). On the other hand, TBS correlated positively with genotype ff *(FokI)* (*r* = 0.63, *p* = 0.04). In the cured acromegaly group, a positive correlation was observed between genotype AA *(ApaI*) and lumbar BMD, femoral neck BMD as well as with TBS (*r* = 0.39, *p* = 0.04; *r* = 0.45, *p* = 0.02; *r* = 0.27, *p* = 0.04, respectively). In whole acromegaly group (AA + CA + CD), as well as in three subgroups (AA, CA, CD) and in the control group, positive correlations were observed between lumbar BMD and femoral neck BMD with TBS. On the other hand, in the same groups, the negative correlations were observed between TBS and risk for major osteoporotic fracture and hip fractures (Table 5).

**Table 5:** Correlations of TBS with lumbar spine BMD, femoral neck BMD, risk of major and hip fractures.

**Groups**	**Lumbar spine BMD**	**Femoral neck BMD**	**Risk of major fracture**	**Risk of hip fracture**
AA+CA+CD	*r* = 0.63, ***p*** **= 0.00**	*r* = 0.48, ***p*** **= 0.00**	*r* = −0.40, ***p*** **= 0.00**	*r* = −0.49, *p* **= 0.00**
AA	*r* = 0.75, ***p*** **= 0.01**	*r* = 0.54, ***p*** **= 0.00**	*r* = −0.41, *p* = 0.13	*r* = −0.52, *p* = 0.07
CA	*r* = 0.8, ***p*** **= 0.00**	*r* = 0.68, ***p*** **= 0.00**	*r* = −0.61, ***p*** **= 0.00**	*r* = −0.67, ***p*** **= 0.00**
CD	*r* = 0.52, ***p*** **= 0.00**	*r* = 0.3, ***p*** **= 0.03**	*r* = −0.29, ***p*** **= 0.03**	*r* = −0.36, ***p*** **= 0.01**
CG	*r* = 0.38, ***p*** **= 0.00**	*r* = 0.26, *p* = 0.06	*r* = −0.56, ***p*** **= 0.00**	*r* = −0.56, ***p*** **= 0.00**

*Bold values statistically significant at p < 0.05*.

**Figure 1:**
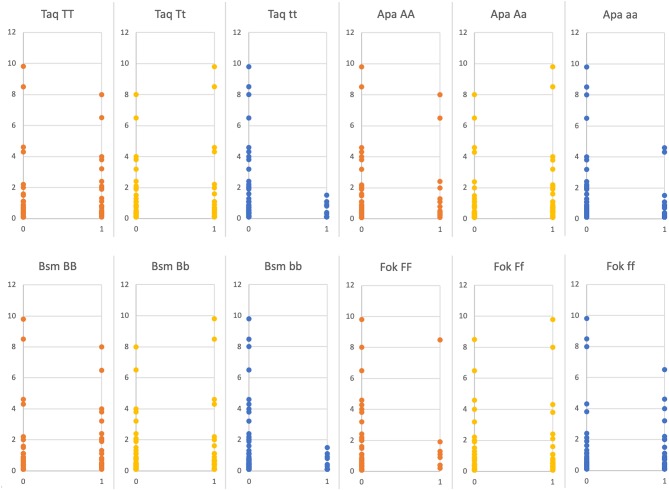
Ten years hip fracture risk FRAX [%] depending on presence (1) or absence (0) of certain *VDR* genes polymorphisms (statistically significant: *TaqI* tt *p* = 0.03; *ApaI* aa *p* = 0.03; *BsmI* bb *p* = 0.03, statistically non-significant *FokI* ff *p* = 0.44).

**Figure 2:**
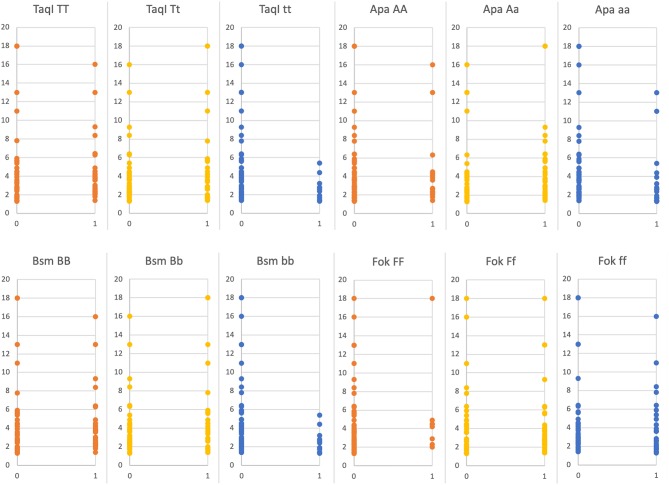
Ten years major osteoporotic fracture risk FRAX [%] depending on presence (1) or absence (0) of certain *VDR* genes polymorphism (statistically significant: *TaqI* tt *p* = 0.01; *ApaI* aa *p* = 0.04; *BsmI* bb *p* = 0.01, statistically non-significant *FokI* ff *p* = 0.21).

The study revealed a statistically significant negative correlation between risk for hip fracture and genotypes tt *(TaqI)*, aa *(ApaI*) and bb (*BsmI*) in acromegaly patients (AA + CA + CD) (*r* = 0.24, *p* = 0.03; *r* = 0.23, *p* = 0.03; *r* = 0.24, *p* = 0.03, respectively) (Figure 1). A similar negative correlation was observed between major risk of osteoporotic fractures and genotypes tt (*TaqI*), aa (*ApaI*) and bb (*BsmI*) in the whole acromegaly group (AA + CA + CD) (*r* = 0.27, *p* = 0.01; *r* = 0.22, *p* = 0.04; *r* = 0.27, *p* = 0.01, respectively) (Figure 2). This negative correlation was also statistically significant in the controlled acromegaly group (CA) for major risk of fractures and genotypes tt (*TaqI*) and bb (*BsmI*) (*r* = 0.38, *p* = 0.00; *r* = 0.38, *p* = 0.00, respectively), as well as risk for hip fracture and genotypes tt (*TaqI*) and bb (*BsmI*) (*r* = 0.4, *p* = 0.00; *r* = 0.4, *p* = 0.00, respectively). What is more, in the CA group, a statistically significant positive correlation was observed between risk of major osteoporotic fractures and genotypes Tt (*TaqI*), Aa (*ApaI*), and Bb (*BsmI*) (*r* = 0.36, *p* = 0.01; *r* = 0.35, *p* = 0.01; *r* = 0.36, *p* = 0.01, respectively), as well as risk for hip fracture and genotypes Tt (*TaqI*), Aa (*ApaI*), and Bb (*BsmI*) (*r* = 0.29, *p* = 0.03; *r* = 0.34, *p* = 0.01; *r* = 0.29, *p* = 0.01, respectively). TBS correlated positively with genotypes tt *(TaqI)*, aa *(ApaI*), and bb (*BsmI*) in acromegaly patients (AA + CA + CD), as well as in AA group and CA group separately, but it was not statistically significant. On the other hand, the tendency for a negative correlation was observed between TBS and genotypes TT, Tt *(TaqI)*, Aa *(ApaI*), and Bb, bb (*BsmI*). In CD group, a statistically significant positive correlation was observed between TBS and genotypes AA *(ApaI)* (*r* = 0.27, *p* = 0.04). Furthermore, in the CA group, a negative correlation was revealed between magnesium level and genotypes TT (*TaqI*), AA (*ApaI*), BB (*BsmI*), and ff (Fok) (*r* = 0.29, *p* = 0.03; *r* = 0.27, *p* = 0.04, *r* = 0.29, *p* = 0.03; *r* = 0.26, *p* = 0.02, respectively).

In the AA group, we found a statistically significant negative correlation between fasting GH levels and genotypes TT (*TaqI*), Aa (*ApaI*), and BB (*BsmI*) (*r* = 0.72, *p* = 0.03; *r* = 0.77, *p* = 0.02; *r* = 0.72, *p* = 0.03, respectively). On the other hand, this study revealed statistically significant positive correlation between fasting GH levels and genotypes tt (*TaqI*), aa (*ApaI*), and bb (*BsmI*) (*r* = 0.72, *p* = 0.03; *r* = 0.77, *p* = 0.02; *r* = 0.72, *p* = 0.03, respectively), as well as between nadir GH and the same genotypes tt (*TaqI*), aa (*ApaI*), and bb (*BsmI*) (*r* = 0.79, *p* = 0.03; *r* = 0.87, *p* = 0.03; *r* = 0.79, *p* = 0.05, respectively).

Moreover, a correlation between *FokI* genotype and hormonal status of acromegaly was found. The patients with the ff genotype showed a statistically significant negative correlation with fasting GH (*r* = 0.24, *p* = 0.02). In the CD group, genotype ff correlated negatively with IGF-1 (*r* = 0.4, *p* = 0.03). In this group, a statistically significant positive correlation between Ff genotype and IGF-1, as well as IGF-1 × ULN, was found (*r* = 0.44, *p* = 0.02; *r* = 0.51, *p* = 0.00, respectively).

## Discussion

The majority of studies showed that the increased level of 1,25(OH) vitamin D in acromegaly is a consequence of the impact of GH on renal vitamin D metabolism, possibly by regulation of activity of 1 alpha-hydroxylase (11). This effect is accompanied by decreased levels of 25 (OH) vitamin D, which may be a consequence of direct effect of GH on liver microsomal enzyme system, caused by number of drugs used in acromegaly (12), as well as the higher BMI in active acromegaly (13). It is known that GH excess is almost always associated with disturbances with vitamin D metabolism, thus, it is postulated that vitamin D should be considered as an useful marker of the GH/IGF-1 system (14). Some studies showed that 1,25(OH) vitamin D3 and its metabolites inhibit human cancer cell growth and proliferation (15, 16). Calcitriol is being evaluated as an anticancer agent in several human cancers. The mechanisms underlying the anticancer properties of calcitriol include anti-proliferative, pro-apoptotic, anti-angiogenic and anti-invasive effect on cells (17, 18). This effect is specially dependent on the activity of vitamin D receptor, which stimulates the expression of more than 200 gene-encoding enzymes responsible for differentiation, proliferation and apoptosis of cells (19, 20). Furthermore, many studies indicate the role of vitamin D and its receptor in processes such as cardiovascular (21) or autoimmune diseases (22). To the best of our knowledge, this is the second report investigating *VDR* polymorphism in acromegaly and first which has assessed the association with risk for osteoporotic fractures in acromegaly.

In our study, we did not find an association between risk of acromegaly and frequencies of these four *VDR* polymorphisms. In one previous study, it was found that the *VDR* FokI ff genotype was associated with a decreased risk and FokI Ff genotype with a significantly increased risk for acromegaly (23). The authors also observed that patients with Ff genotype had higher IGF-1 level after treatment compared to patients with ff genotype. In our study, we revealed a negative correlation between fasting GH and FokI ff genotype in all acromegaly groups and with IGF-1 levels in the cured disease group. What is more, in CD group, a positive correlation between Ff genotypes and IGF-1, as well as with IGF-1 according to the upper limit of normal, was demonstrated. It is known that f allele corresponds with the 427 aa long VDR protein variant and less transcriptional activity, as the F allele results in a shorter and more active variant (6). In the AA group, we also found a statistically significant negative correlation between fasting GH levels and genotypes TT (*TaqI*), Aa (*ApaI*), and BB (*BsmI*) and a positive correlation between fasting GH levels and nadir GH with genotypes tt (*TaqI*), aa (*ApaI*), and bb (*BsmI*). These findings may suggest a possible role of these polymorphisms in the course of acromegaly as a consequence of altering hormonal status. These polymorphisms are located on the 3′ regulatory area, and effects on mRNA stability (24).

In this study, we did not observe differences in the mean values of 25(OH)D3 among all tested groups. In addition, the correlation between 25(OH)D3 levels and parameters of calcium, magnesium and phosphate homeostasis, as well as risk for osteoporotic fractures, were not found. The role of vitamin D in acromegaly is not clear. Some of the studies showed increased vitamin D levels in acromegaly, especially 1,25(OH) D3 (11, 25), and other studies revealed low vitamin D in this group of patients (13). In our study, a lack of difference may be the result of supplementation of vitamin D in the acromegaly group. We observed a negative correlation between major risk osteoporotic fractures, as well as risk for hip fracture and genotypes tt (*TaqI*), aa (*ApaI*), and bb (*BsmI*) in acromegaly group and in the cured disease group, as well as a positive correlation with genotypes Tt (*TaqI*), Aa (*ApaI*), and Bb (*BsmI*). Results on the relationships between vitamin D receptor (*VDR*) gene polymorphisms and postmenopausal osteoporosis susceptibility and BMD are conflicting. Some of them revealed the statistically significant association with the risk of developing postmenopausal osteoporosis in women with BB and Bb (*BsmI*) and Aa (*ApaI*) genotypes. No significant relationship was observed between *VDR Taq*I polymorphism and postmenopausal osteoporosis susceptibility in most of the studies (26), but Ahmad et al. revealed that the *TaqI* gene TT genotype was associated with low BMD in North Indian osteoporotic women (27).

Patients with acromegaly have an increased risk of fractures, which might be correlated with insufficient quality of bone (28). More importantly, vertebral fractures may be present in patients with normal or slightly decreased BMD, which makes BMD a poor fracture predictor (28–30). It is known that the use of FRAX in acromegaly is not validated, however we don't have enough other useful tools to assess real risk for fractures. It is known that it may not represent the actual risk for vertebral fractures. The early diagnosis of acromegaly, as well as investigations for new markers of risk for osteoporotic fractures, seem to be the most important tools in reducing this risk. The valuable tool in the assessment of bone structure is the TBS measurement to obtain some surrogate information on bone microarchitecture from a routine DXA. The high TBS score reflects better bone structure, whereas a low TBS score indicates impaired structures that is prone to fractures (31). Low TBS values have been shown in patients with acromegaly and vertebral fractures (32). In our study, we did not observe a statistically significant difference in TBS values between the acromegaly group and the control group. Furthermore, we observed a positive correlation between TBS and BMD, and even more important to note: the negative correlation found with the risk of major and hip fractures in acromegalic patients. TBS values, similarly to BMD, correlated negatively with genotypes Ff *(FokI)* and positively with genotypes ff *(FokI)* in AA group. The tendency for a positive correlation was also observed between TBS values with genotypes tt *(TaqI)*, aa *(ApaI*), and bb (*BsmI*) in acromegaly patients. This was not statistically significant but consistent with the negative relation of the risk of fractures and these polymorphisms. Despite a relatively small sample size, it has been shown that analyzed polymorphisms may be associated with variable bone mass and risk of fractures in patients with acromegaly. This can lead to reduced risk of fractures and better bone quality in patients with acromegaly, who are protective-allele carriers.

There are some limitations to this study. First, the sample size of the active acromegaly group, as well as group of all acromegaly patients, was small and the results of this study should be analyzed in a larger group. Secondly, the various duration of the disease, different dosage and time span of therapy with somatostatin analog may have had an impact on the results. Finally, different supplementation of daily vitamin D and bisphosphonate therapy in acromegaly and control groups may influence the results of this study.

In conclusion, these results may suggest a possible role of the *FokI* ff genotype in the effectiveness of acromegaly treatment, as well as with better quality of bone mass. TBS may be a useful tool for predicting risk of fractures in acromegaly patients. What is more, tt (*TaqI*), aa (*ApaI*), and bb (*BsmI*) genotypes of *VDR* gene may be associated with better bone quality and microarchitecture (higher TBS), which may lead to a lower risk of osteoporotic fractures in acromegaly patients. Nevertheless, further studies are needed to elucidate these findings.

## Data Availability Statement

The datasets generated for this study can be found in dbSNP (http://www.ncbi.nlm.nih.gov/snp).

## Ethics Statement

This study was carried out in accordance with the recommendations of local Bioethics Committee Medical University, Wroclaw. The protocol was approved by the local Bioethics Committee of Medical University, Wroclaw, Poland. All subjects gave written informed consent in accordance with the Declaration of Helsinki.

## Author Contributions

AJ-P designed the project, the main conceptual ideas and proof outline, interpretation of the results, complied the literature sources, wrote manuscript, and checked the references. JH-Ż and MB contributed conception and design of the study, helped in collection date, their interpretation, and reference checking. KK and AZ performed laboratory measurement and wrote section of the manuscript. ŁG was responsible for statistical analysis and designed the figures, help in date interpretation. All authors contributed to the final version of the manuscript and approved it for publication.

### Conflict of Interest

The authors declare that the research was conducted in the absence of any commercial or financial relationships that could be construed as a potential conflict of interest.
